# 1,4-Bis(pyridin-3-ylmeth­oxy)benzene

**DOI:** 10.1107/S1600536809036265

**Published:** 2009-09-12

**Authors:** Jin-Sheng Gao, Ying Liu, Shuang Zhang, Dian-Fa Zuo, Guang-Feng Hou

**Affiliations:** a College of Chemistry and Materials Science, Heilongjiang University, Harbin 150080, People’s Republic of China

## Abstract

The asymmetric unit of the centrosymmetric title compound, C_18_H_16_N_2_O_2_, contains one half-mol­ecule. The central benzene ring forms a dihedral angle of 66.8 (1)° with two outer aromatic rings. In the crystal structure, weak inter­molecular C—H⋯N hydrogen bonds link mol­ecules into sheets parallel to (104).

## Related literature

For general background to bridging mol­ecules with pyridyl substituents at the terminal positions, see: McMorran & Steel (1998 ▶); Zaman *et al.* (2005 ▶). For details of the synthesis, see: Gao *et al.* (2004 ▶). For a related structure, see: Gao *et al.* (2006 ▶).
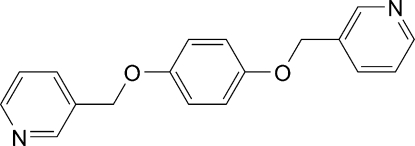


## Experimental

### 

#### Crystal data

C_18_H_16_N_2_O_2_
*M* *_r_* = 292.33
Monoclinic, 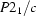

*a* = 6.852 (5) Å
*b* = 5.688 (3) Å
*c* = 18.861 (12) Å
β = 90.60 (3)°
*V* = 735.0 (8) Å^3^
*Z* = 2
Mo *K*α radiation
μ = 0.09 mm^−1^
*T* = 291 K
0.22 × 0.20 × 0.19 mm

#### Data collection

Rigaku RAXIS-RAPID diffractometer
Absorption correction: multi-scan (*ABSCOR*; Higashi, 1995 ▶) *T*_min_ = 0.981, *T*_max_ = 0.984
6855 measured reflections
1684 independent reflections
1213 reflections with *I* > 2σ(*I*)
*R*_int_ = 0.030

#### Refinement

*R*[*F* ^2^ > 2σ(*F* ^2^)] = 0.041
*wR*(*F* ^2^) = 0.118
*S* = 1.09
1684 reflections
100 parameters
H-atom parameters constrained
Δρ_max_ = 0.24 e Å^−3^
Δρ_min_ = −0.14 e Å^−3^

### 

Data collection: *RAPID-AUTO* (Rigaku, 1998 ▶); cell refinement: *RAPID-AUTO*; data reduction: *CrystalClear* (Rigaku/MSC, 2002 ▶); program(s) used to solve structure: *SHELXS97* (Sheldrick, 2008 ▶); program(s) used to refine structure: *SHELXL97* (Sheldrick, 2008 ▶); molecular graphics: *SHELXTL* (Sheldrick, 2008 ▶); software used to prepare material for publication: *SHELXL97*.

## Supplementary Material

Crystal structure: contains datablocks I. DOI: 10.1107/S1600536809036265/cv2613sup1.cif

Structure factors: contains datablocks I. DOI: 10.1107/S1600536809036265/cv2613Isup2.hkl

Additional supplementary materials:  crystallographic information; 3D view; checkCIF report

## Figures and Tables

**Table 1:** Hydrogen-bond geometry (Å, °)

*D*—H⋯*A*	*D*—H	H⋯*A*	*D*⋯*A*	*D*—H⋯*A*
C1—H1⋯N1^i^	0.93	2.57	3.437 (3)	155

Symmetry code: (i) 
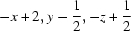
.

**Fig. 1.**
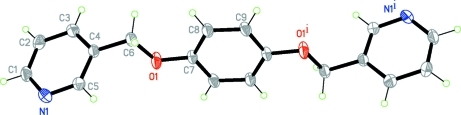
The molecular structure of the title compound, showing the atomic numbering and displacement ellipsoids at the 30% probability level [symmetry code: (i) -x, 1 - y, -z].

**Fig. 2.**
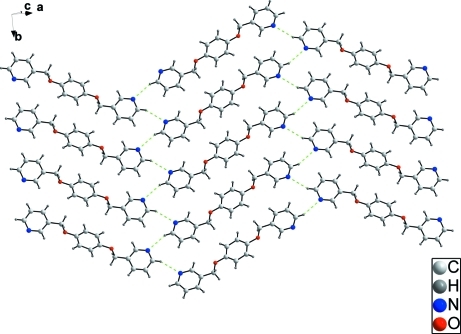
A portion of the crystal packing showing the two-dimensional hydrogen bonded (dashed lines) sheet.

## supplementary crystallographic information

### Comment

The bridging molecules with pyridyl substituents at the terminal positions are hoped to construct interesting supramolecular architectures by intermolecular hydrogen bonding and coordination with metals. McMorran' group have reported the synthesis of a quadruply stranded helicate that encapsulates a hexafluorophosphate anion by the reaction of 1,4-bis(3-pyridylmethoxy)benzene with palladium chlorate (McMorran *et al.*, 1998). Zaman's group have designed a long rigid organic ligand, 1,4-bis[(3-pyridyl)ethynyl]benzene, which reacted with metal salts to form interpenetrating two-dimensional and three-dimensional cross-zigzag chains and metallocyclic chain structures (Zaman *et al.*, 2005). As an extension of our work about bipyridyl aromatic ligands, we report the crystal structure of the title compound here.

In the title compound (Fig. 1), all bond lengths and angles are normal and correpond to those observed in the related compound (Gao *et al.*, 2006). The 1,4-bis(3-pyridylmethoxy)benzene molecule is centrosymmetric. The planes of two terminal pyridyl groups rotate drastically and make dihedral angles of 66.8 (1) ° with the plane of the central benzene ring.

In the crystal structure, the adjacent 1,4-bis(3-pyridylmethoxy)benzene molecules are linked into two-dimensional supramolecular sheets by intermolecular C—H···N hydrogen bonds (Table 1, Figure 2).

### Experimental

The 1,4-bis(3-pyridylmethoxy)benzene was synthesized by the reaction of *p*-benzenediol and 3-chloromethylpyridine hydrochloride under nitrogen atmosphere and alkaline condition (Gao *et al.*, 2004; Gao *et al.*, 2006). Colourless block-shaped crystals of title compound were obtained by slow evaporation of an methanol solution after three days.

### Refinement

All H atoms were placed in calculated positions with C—H = 0.93 Å (aromatic), C—H = 0.97 Å (methylene), and treated as riding on their parent atoms, with *U*_iso_(H) = 1.2*U*_eq_(C).

### Crystal data

**Table:**

C_18_H_16_N_2_O_2_	*F*(000) = 308
*M**_r_* = 292.33	*D*_x_ = 1.321 Mg m^−^^3^
Monoclinic, *P*2_1_/*c*	Mo *K*α radiation, λ = 0.71073 Å
Hall symbol: -P 2ybc	Cell parameters from 4889 reflections
*a* = 6.852 (5) Å	θ = 3.7–27.5°
*b* = 5.688 (3) Å	µ = 0.09 mm^−^^1^
*c* = 18.861 (12) Å	*T* = 291 K
β = 90.60 (3)°	Block, colourless
*V* = 735.0 (8) Å^3^	0.22 × 0.20 × 0.19 mm
*Z* = 2	

### Data collection

**Table:**

Rigaku RAXIS-RAPID diffractometer	1684 independent reflections
Radiation source: fine-focus sealed tube	1213 reflections with *I* > 2σ(*I*)
graphite	*R*_int_ = 0.030
ω scan	θ_max_ = 27.5°, θ_min_ = 3.7°
Absorption correction: multi-scan (*ABSCOR*; Higashi, 1995)	*h* = −8→8
*T*_min_ = 0.981, *T*_max_ = 0.984	*k* = −6→7
6855 measured reflections	*l* = −24→24

### Refinement

**Table:**

Refinement on *F*^2^	Primary atom site location: structure-invariant direct methods
Least-squares matrix: full	Secondary atom site location: difference Fourier map
*R*[*F*^2^ > 2σ(*F*^2^)] = 0.041	Hydrogen site location: inferred from neighbouring sites
*wR*(*F*^2^) = 0.118	H-atom parameters constrained
*S* = 1.09	*w* = 1/[σ^2^(*F*_o_^2^) + (0.0606*P*)^2^ + 0.0635*P*] where *P* = (*F*_o_^2^ + 2*F*_c_^2^)/3
1684 reflections	(Δ/σ)_max_ < 0.001
100 parameters	Δρ_max_ = 0.24 e Å^−^^3^
0 restraints	Δρ_min_ = −0.14 e Å^−^^3^

### Special details

**Table:**

Geometry. All e.s.d.'s (except the e.s.d. in the dihedral angle between two l.s. planes) are estimated using the full covariance matrix. The cell e.s.d.'s are taken into account individually in the estimation of e.s.d.'s in distances, angles and torsion angles; correlations between e.s.d.'s in cell parameters are only used when they are defined by crystal symmetry. An approximate (isotropic) treatment of cell e.s.d.'s is used for estimating e.s.d.'s involving l.s. planes.
Refinement. Refinement of *F*^2^ against ALL reflections. The weighted *R*-factor *wR* and goodness of fit *S* are based on *F*^2^, conventional *R*-factors *R* are based on *F*, with *F* set to zero for negative *F*^2^. The threshold expression of *F*^2^ > σ(*F*^2^) is used only for calculating *R*-factors(gt) *etc*. and is not relevant to the choice of reflections for refinement. *R*-factors based on *F*^2^ are statistically about twice as large as those based on *F*, and *R*- factors based on ALL data will be even larger.

### Fractional atomic coordinates and isotropic or equivalent isotropic displacement parameters (Å^2^)

**Table:**

	*x*	*y*	*z*	*U*_iso_*/*U*_eq_	
C1	0.8460 (2)	−0.0288 (3)	0.20865 (8)	0.0485 (4)	
H1	0.9650	−0.0611	0.2307	0.058*	
C2	0.7721 (2)	−0.1906 (3)	0.16191 (8)	0.0530 (4)	
H2	0.8404	−0.3281	0.1524	0.064*	
C3	0.5951 (2)	−0.1469 (3)	0.12921 (8)	0.0484 (4)	
H3	0.5422	−0.2545	0.0972	0.058*	
C4	0.49744 (18)	0.0588 (2)	0.14460 (7)	0.0367 (3)	
C5	0.5857 (2)	0.2119 (3)	0.19208 (7)	0.0428 (4)	
H5	0.5213	0.3517	0.2022	0.051*	
C6	0.30072 (19)	0.1158 (3)	0.11378 (7)	0.0433 (4)	
H6A	0.2437	−0.0233	0.0922	0.052*	
H6B	0.2144	0.1704	0.1507	0.052*	
C7	0.15874 (17)	0.3913 (2)	0.03246 (7)	0.0368 (3)	
C8	−0.02824 (18)	0.3034 (3)	0.04198 (7)	0.0408 (3)	
H8	−0.0477	0.1710	0.0700	0.049*	
C9	−0.18583 (18)	0.4142 (3)	0.00955 (7)	0.0408 (3)	
H9	−0.3112	0.3563	0.0163	0.049*	
N1	0.75686 (17)	0.1729 (2)	0.22431 (7)	0.0510 (4)	
O1	0.32526 (13)	0.29466 (19)	0.06180 (5)	0.0509 (3)	

### Atomic displacement parameters (Å^2^)

**Table:**

	*U*^11^	*U*^22^	*U*^33^	*U*^12^	*U*^13^	*U*^23^
C1	0.0308 (7)	0.0572 (9)	0.0571 (9)	0.0002 (6)	−0.0101 (6)	0.0171 (7)
C2	0.0439 (9)	0.0449 (8)	0.0700 (10)	0.0117 (7)	−0.0063 (7)	0.0064 (8)
C3	0.0487 (8)	0.0432 (8)	0.0532 (8)	0.0035 (7)	−0.0108 (6)	−0.0038 (7)
C4	0.0308 (6)	0.0395 (7)	0.0396 (7)	−0.0005 (5)	−0.0056 (5)	0.0083 (6)
C5	0.0371 (7)	0.0388 (7)	0.0525 (8)	0.0016 (6)	−0.0069 (6)	0.0008 (6)
C6	0.0334 (7)	0.0482 (8)	0.0482 (8)	−0.0010 (6)	−0.0093 (6)	0.0109 (6)
C7	0.0264 (6)	0.0465 (8)	0.0374 (6)	0.0056 (5)	−0.0046 (5)	0.0038 (6)
C8	0.0308 (7)	0.0475 (8)	0.0438 (7)	−0.0007 (6)	−0.0045 (5)	0.0111 (6)
C9	0.0250 (6)	0.0530 (8)	0.0444 (7)	−0.0016 (6)	−0.0030 (5)	0.0073 (6)
N1	0.0394 (7)	0.0538 (8)	0.0596 (8)	−0.0056 (6)	−0.0145 (6)	0.0004 (6)
O1	0.0269 (5)	0.0683 (7)	0.0573 (6)	0.0058 (4)	−0.0048 (4)	0.0267 (5)

### Geometric parameters (Å, °)

**Table:**

C1—N1	1.334 (2)	C6—O1	1.4239 (17)
C1—C2	1.368 (2)	C6—H6A	0.9700
C1—H1	0.9300	C6—H6B	0.9700
C2—C3	1.378 (2)	C7—C9^i^	1.374 (2)
C2—H2	0.9300	C7—O1	1.3773 (17)
C3—C4	1.380 (2)	C7—C8	1.389 (2)
C3—H3	0.9300	C8—C9	1.3869 (19)
C4—C5	1.3839 (19)	C8—H8	0.9300
C4—C6	1.4979 (19)	C9—C7^i^	1.374 (2)
C5—N1	1.3336 (19)	C9—H9	0.9300
C5—H5	0.9300		
			
N1—C1—C2	123.64 (13)	O1—C6—H6A	110.1
N1—C1—H1	118.2	C4—C6—H6A	110.1
C2—C1—H1	118.2	O1—C6—H6B	110.1
C1—C2—C3	119.03 (14)	C4—C6—H6B	110.1
C1—C2—H2	120.5	H6A—C6—H6B	108.4
C3—C2—H2	120.5	C9^i^—C7—O1	115.88 (11)
C2—C3—C4	119.04 (14)	C9^i^—C7—C8	119.64 (12)
C2—C3—H3	120.5	O1—C7—C8	124.47 (13)
C4—C3—H3	120.5	C9—C8—C7	119.64 (14)
C3—C4—C5	117.37 (13)	C9—C8—H8	120.2
C3—C4—C6	122.56 (13)	C7—C8—H8	120.2
C5—C4—C6	120.04 (13)	C7^i^—C9—C8	120.71 (12)
N1—C5—C4	124.50 (14)	C7^i^—C9—H9	119.6
N1—C5—H5	117.7	C8—C9—H9	119.6
C4—C5—H5	117.7	C5—N1—C1	116.41 (13)
O1—C6—C4	108.04 (11)	C7—O1—C6	117.28 (10)

Symmetry codes: (i) −*x*, −*y*+1, −*z*.

### Hydrogen-bond geometry (Å, °)

**Table:**

*D*—H···*A*	*D*—H	H···*A*	*D*···*A*	*D*—H···*A*
C1—H1···N1^ii^	0.93	2.57	3.437 (3)	155

Symmetry codes: (ii) −*x*+2, *y*−1/2, −*z*+1/2.
